# Correction: Zebrafish larvae exposed to ginkgotoxin exhibit seizure-like behavior that is relieved by pyridoxal-5′-phosphate, GABA and anti-epileptic drugs

**DOI:** 10.1242/dmm.050550

**Published:** 2023-10-20

**Authors:** Gang-Hui Lee, Shian-Ying Sung, Wen-Ni Chang, Tseng-Ting Kao, Hung-Chi Tu, Tsun-Hsien Hsiao, Martin K. Safo, Tzu-Fun Fu

There was an error in *Dis. Model. Mech.* (2012) **5**, 785-795 (doi:10.1242/dmm.009449).

The name of the author Hung-Chi Tu was spelled incorrectly (Hung-Chi Du). The correct name appears above, and the author contributions have been updated.

The authors apologise for this error and any inconvenience it may have caused. Both the online full-text and PDF versions of the article have been updated.

